# Anti-Larval and Anti-Algal Natural Products from Marine Microorganisms as Sources of Anti-Biofilm Agents

**DOI:** 10.3390/md20020090

**Published:** 2022-01-21

**Authors:** Kai-Ling Wang, Zheng-Rong Dou, Gao-Fen Gong, Hai-Feng Li, Bei Jiang, Ying Xu

**Affiliations:** 1 Yunnan Key Laboratory of Screening and Research on Anti-Pathogenic Plant Resources from West Yunnan (Cultivation), Institute of Materia Medica, College of Pharmacy, Dali University, Dali 671000, China; kailingw@dali.edu.cn (K.-L.W.); zhengrongdou2020@163.com (Z.-R.D.); gaofen2020@163.com (G.-F.G.); lihfzh888@sina.com (H.-F.L.); jiangbei@dali.edu.cn (B.J.); 2 Shenzhen Key Laboratory of Marine Bioresource & Eco-Environmental Science, Shenzhen Engineering Laboratory for Marine Algal Biotechnology, College of Life Sciences and Oceanography, Shenzhen University, Shenzhen 518060, China

**Keywords:** biofouling, anti-biofilm, anti-larval, anti-algal, antifouling, marine microorganisms, natural products

## Abstract

Bacteria growing inside biofilms are more resistant to hostile environments, conventional antibiotics, and mechanical stresses than their planktonic counterparts. It is estimated that more than 80% of microbial infections in human patients are biofilm-based, and biofouling induced by the biofilms of some bacteria causes serious ecological and economic problems throughout the world. Therefore, exploring highly effective anti-biofilm compounds has become an urgent demand for the medical and marine industries. Marine microorganisms, a well-documented and prolific source of natural products, provide an array of structurally distinct secondary metabolites with diverse biological activities. However, up to date, only a handful of anti-biofilm natural products derived from marine microorganisms have been reported. Meanwhile, it is worth noting that some promising antifouling (AF) compounds from marine microbes, particularly those that inhibit settlement of fouling invertebrate larvae and algal spores, can be considered as potential anti-biofilm agents owing to the well-known knowledge of the correlations between biofilm formation and the biofouling process of fouling organisms. In this review, a total of 112 anti-biofilm, anti-larval, and anti-algal natural products from marine microbes and 26 of their synthetic analogues are highlighted from 2000 to 2021. These compounds are introduced based on their microbial origins, and then categorized into the following different structural groups: fatty acids, butenolides, terpenoids, steroids, phenols, phenyl ethers, polyketides, alkaloids, flavonoids, amines, nucleosides, and peptides. The preliminary structure-activity relationships (SAR) of some important compounds are also briefly discussed. Finally, current challenges and future research perspectives are proposed based on opinions from many previous reviews.

## 1. Introduction

Bacterial biofilms are surface, or interphase-attached microorganism communities that are encapsulated in a self-secreted extracellular matrix comprising largely of proteins, polysaccharides, nucleic acids, and lipids [1,2]. Biofouling, defined as the undesirable growth of micro- and macro-organisms on submerged surfaces, causes serious ecological and economic problems throughout the world [3]. In natural seawater, all substrata are quickly fouled by marine fouling organisms, which is a complex process [4,5]. During the biofouling process, the formation of a biofilm acts as the glue that binds macro-foulers to the surface [2,5,6]. Biofilm formation consisting of microbes and microalgae is often the first step in the progression of biofouling development and is also increasingly recognized to play an essential role in the colonization of marine macro-foulers [5,6,7,8]. Within biofilms, microorganisms interact with a settlement of larvae of fouling invertebrates and algal spores [7,9].

Microbial biofilms formed by certain kinds of pathogenic and fouling bacteria play an adverse role in the medical and marine industries. Over 80% of microbial infections in the body are induced by biofilms [10]. The CDC declares that biofilms have become one of the most important medical hurdles of the century [11]. At the same time, it should be noted that biofouling based on the biofilm formation of fouling microbes causes enormous economic losses annually to the maritime and medical industries [2,5,6,7,8,9,10,11]. There is an urgent demand for exploring effective anti-biofilm agents to combat biofilms. Marine microorganisms, owing to their unique living environment, are extremely rich in natural products with diverse biological activities [12]. However, to our best knowledge, only a handful of anti-biofilm secondary metabolites of marine microbes and their mechanisms of action have been reported in the past few decades [13,14,15]. A massive amount of genomic and proteomic data generated allowing for further illustrations of the unknown molecular mechanisms in the association of biofilm formation and drug resistance [14,15]. The present review primarily focuses on modes of action of some anti-biofilm compounds via interfering with the quorum-sensing pathways, the disruption of extracellular polymeric substances, and adhesion mechanisms reported in the literature from 2000 to 2021.

As mentioned in many previous reviews [5,6,7,9], the development of a biofilm on a substratum could attract the biofouling of marine invertebrates (such as the barnacle *Balanus amphitrite*, the bryozoan *Bugula neritina*, the polychaete *Hydroides elegans*, the mussel *Mytilus coruscus,* and so on) and algae through physically modifying surfaces and releasing chemical compounds. That means numerous antifouling (AF) compounds, especially some environmentally friendly anti-larval/anti-algal agents with an EC_50_ value of <5 μg/mL and a LC_50_/EC_50_ ratio of >50, could serve as a potential source for the exploitation of highly effective and low- or non-toxic anti-biofilm candidates. More encouragingly, in the past two decades, marine microorganisms have provided a large number of potential AF natural products, some of which can be considered a promising source for the further exploration of potent anti-biofilm agents according to some previous reviews [5,7,16,17,18,19,20].

This review focuses on the anti-biofilm, anti-larval, and anti-algal secondary metabolites of marine microorganisms from 2000 up to the end of 2021. A total of 112 important active natural products, including 12 anti-biofilm compounds and 122 AF compounds, together with their 26 synthetic analogs, are described and highlighted with examples. Meanwhile, the preliminary analysis of the structure-relationship (SAR) of some compounds is discussed briefly. All selected compounds are sequentially introduced based on their chemical structure types in the relevant sections of this review. The further direction in the search for anti-biofilm/AF natural products from marine microbes is prosed in the concluding remarks.

## 2. Anti-Biofilm and Antifouling Natural Products from Marine Bacteria

**Figure 1:**
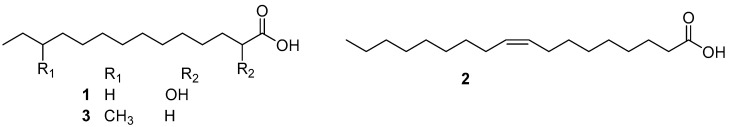
Chemical structures of fatty acids **1** and **2** marine bacteria *Shewanella oneidensis* SCH0402**,** and 12-methyltetradecanoid acid (**3**) from *Streptomyces* sp.

**Figure 2:**
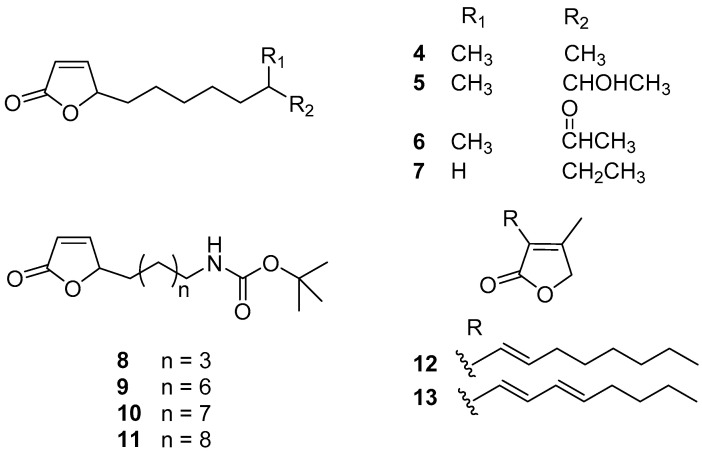
Chemical structures of butenolides **4**–**13**. Among them, compounds **4**–**6** were isolated from marine bacteria *Streptomyces albidoflavus* UST040711-291 and *Streptomyces* sp. GWS-BW-H5, compounds **7**–**11** are synthetic ones, and compounds **12** and **13** were isolated from a marine bacterium *S. violaceoruber* SCH-09.

**Figure 3:**
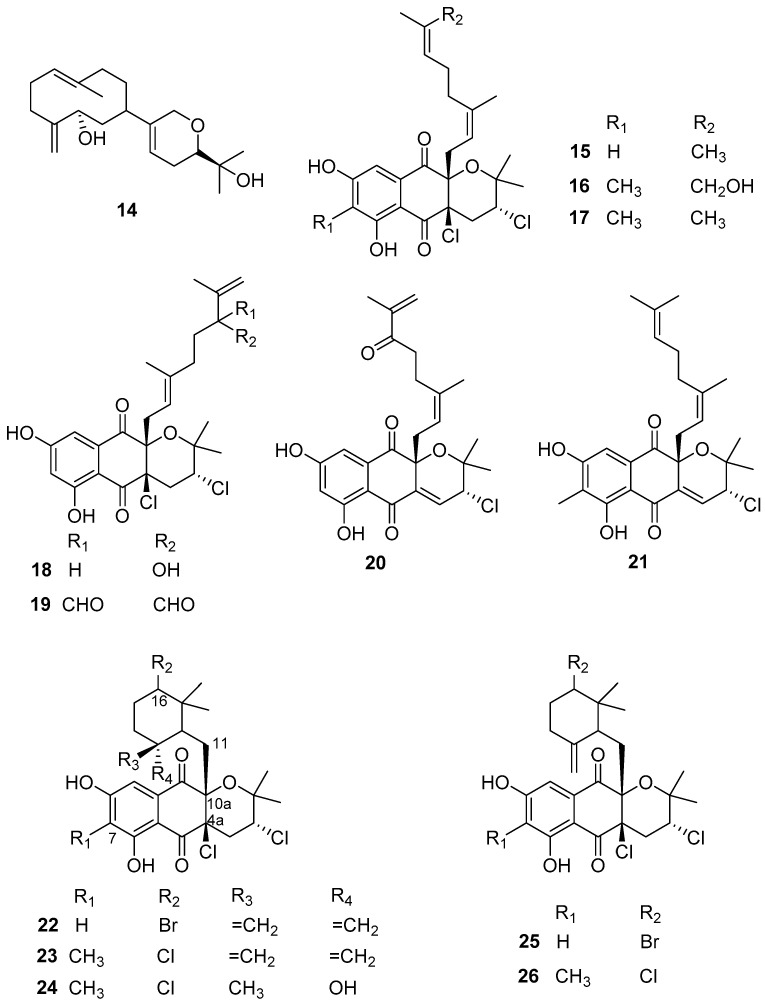
Chemical structures of terpenoids **14**–**26** from actinobacterial species of *Streptomyces.* Among them, the diterpene compound **14** was isolated from *S. cinnabarinus* PK209, the napyradiomycins **15**, **16**, **18**–**20**, **22** and **25** having a hydrogen atom at C-7 in the semi-naphthoquinone core structure were obtained from *S. aculeolatus* strain PTM-420, and another napyradiomycin compounds **17**, **21**, **23**, **24** and **26** containing methyl group at C-7 were obtained from *S. aculeolatus* strain PTM-029.

**Figure 4:**
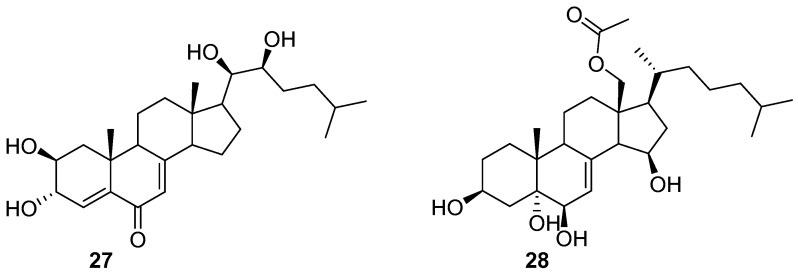
Chemical structures of steroid compound **27** and **28** from a filamentous bacterium *Leucothrix mucor*.

**Figure 5:**
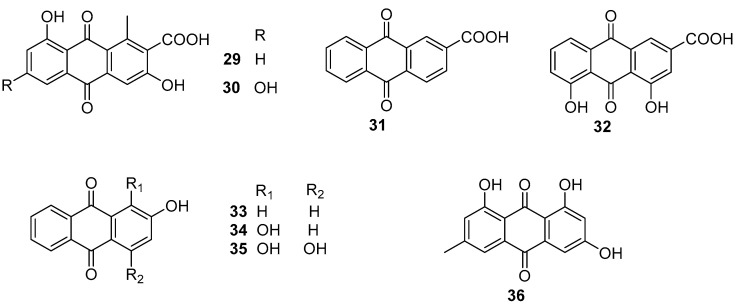
Chemical structures of anthraquinones **29**–**36** from a rare actinobacterium *Kitasatospora* *albolonga* R62.

**Figure 6:**
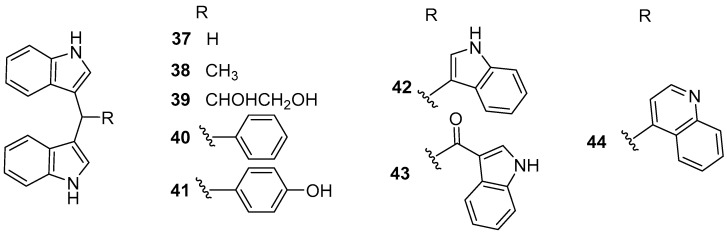
Chemical structures of bisindole alkaloids **37**–**44** from a marine bacterium *Pseudovibrio denitrificans* UST4-50.

**Figure 7:**
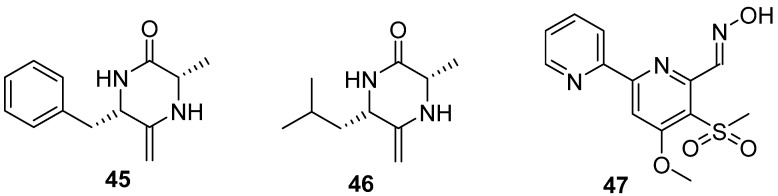
Chemical structures of diketopiperazine alkaloids **45** and **46** from Streptomyces praecox 291-11, and maipomycin A **47** from a rare actinomycete *Kibdelosporangium phytohabitans* XY-R10.

**Figure 8:**
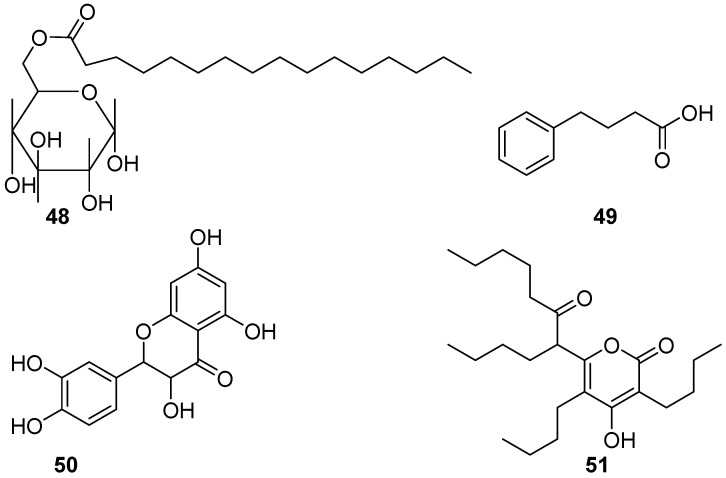
Chemical structures of the glycolipid surfactant **48** from a marine strain of *Serratia marcescens*, the benzenoid compound 4-phenylbutanoic acid (**49**) from a bacterium *Bacillus pumilus* strain S6-15, the flavonoid compound taxifolin (**50**) from actinobacterial *Streptomyces sampsonii* PM33, and the polyketide compound elasnin (**51**) from *S. mobaraensis* DSM 40847.

Marine bacteria are well-recognized as the largest microbial group in the marine environment. Several anti-larval and anti-algal natural products have been isolated from diverse marine bacteria, including the species of the bacterial genera *Shewanella*, *Pseudovibrio*, *Pseudoalteromonas*, and *Pseudomonas*, and of the actinobacterial genus *Streptomyces* [21,22,23,24,25,26,27,28,29,30,31,32,33,34,35,36,37,38,39,40,41,42]. All secondary metabolites of these marine bacteria are described based on their chemical structures, including the following: fatty acids, butenolides, terpenoids, steroids, alkaloids, glycolipids, benzenoids, flavonoids, polyketides, and enzymes (Figure 1, Figure 2, Figure 3, Figure 4, Figure 5, Figure 6, Figure 7 and Figure 8).

### 2.1. Fatty Acids

Isolation of AF compounds from the chloroform extract of a marine bacterium, *Shewanella oneidensis* SCH0402, yielded two fatty acids (*S*)-2-hydroxymyristic acid (HMA) (**1**) and cis-9-oleic acid (COA) (**2**) (Figure 1). During the one-and-a-half-year period of the field trial, both displayed inhibition for the germination of the representative soft fouling macro-algae *Ulva pertusa* spores at respective concentrations of 10 and 100 μg/mL, and strong AF activity as potent as TBTO against a wide range of micro- and macro-foulers. More importantly, these two fatty acids can be completely biodegraded, suggesting that this class of compounds can be used as environmentally friendly substitutes for toxic antifoulants [21]. Similarly, as an HMA-like homolog, 12-methyltetradecanoid acid (12-MTA) (**3**) (Figure 1), obtained from a deep-sea *Streptomyces* sp. through bioassay-guided isolation procedure, was found to have effective inhibition on larval settlement of *H. elegans* with EC_50_ value of 0.6 μg/mL, while its toxicity was very low with a high LC_50_/EC_50_ ratio of >133.5. Further study on AF mechanism revealed that the inhibitory effects of 12-MTA on the settlement of *H. elegans* larvae might be related to the down-regulation of GTPase-activating gene and up-regulation of ATP synthase gene [22].

### 2.2. Butenolides

Actinobacteria is the largest phylum in the bacterial domain and has great potential for producing novel and bioactive natural products including antifoulants. A series of butenolides (Figure 2) were isolated from two actinobacterial strains, a deep-sea derived *Streptomyces albidoflavus* UST040711-291 and a North Sea derived *Streptomyces* sp. GWS-BW-H5. Among these butenolides, compounds **4**–**6** prevented larval settlement of *B. amphitrite* at concentrations less than 10.00 μg/mL, and their toxicity was weak [23,24]. Analysis of SAR revealed that 2-furanone ring, the common moiety, may be essential for AF activity, while the lipophilicity of the alkyl side-chain linked to the mother nucleus may explain their varying potency of AF activity. Based on these findings, compound **7** (5-octylfuran-2(5H)-one)**,** named butenolide, structured with a straight alkyl chain exhibited significant and non-toxic anti-larval activity against settlement of *B. amphitrite*, *B. neritina,* and *H. elegans* larvae with EC_50_ values of 0.518, 0.199, and 0.0168 μg/mL and LC_50_/EC_50_ ratios higher than 97, 250, and 119, respectively. More importantly, this compound also displayed outstanding AF activity even at a concentration of 5% (*w*/*w*) in field tests, indicating that it might be a promising candidate for AF purposes [24]. Because of the excellent AF activity of butenolide, a dozen analogues of butenolide were synthesized for AF bioassays, among which the Boc-butenolide (**8**) structurally modified with a Boc-protecting-group at the terminal of the alkyl side-chain was demonstrated to have similar AF capabilities to butenolide in larval settlement bioassays but with significantly lower toxicity at high concentration of 25 μg/mL. Meanwhile, Boc-butenolide has good environmental stability with a higher melting point at 132 °C higher than that of butenolide at 23 °C. The coverage of biofouler on the Boc-butenolide coatings was low after 2 months in a marine field test, indicating the AF potential of Boc-butenolide [25]. The other three synthesized butenolide derivatives **9**–**11** showed significant inhibition for larval settlement of *B. amphitrite* with EC_50_ values of 0.663, 0.722, and 0.827 μg/mL, respectively, whereas they were less toxic with the LC_50_/EC_50_ ratios >61, 73, and 63, respectively [26]. In addition, another two known natural butenolides, compounds **12** and **13**, were isolated from a seaweed epibiotic bacterium *S. violaceoruber* SCH-09. Both of them displayed strong anti-larval activity against the mussel *M. edulis* with EC_50_ values of 0.02 and 0.1 μg/mL, while their toxicity is very low with high therapeutic ratios >92 [27]. Moreover, based on the investigations of mode of AF action from proteome and phosphoproteome profiles, Zhang et al. [28] and Qian et al. [29] suggested that butenolide prevented larval settlement of macro-fouling organisms *B. amphitrite* and *B. neritina* by modulating proteins involved in stress regulation and energy metabolism in larvae.

Recently, Ding et al. [30] reported anti-biofilm activity of this compound against mixed marine bacterial species in a field experiment. After one month, the panels coated with paints containing 10% (*w*/*w*) of butenolide were free of biofilm formation, and only a small amount of biofilm occurred on the panels with 2.5 and 5% of butenolide. According to the results of metatranscriptomic analysis, the microbial community structure of biofilm was visualized at the phylum level, including Bacteroidetes/Chlorobi, Chloroflexi, Gammaproteobacteria, Cyanobacteria, Actinobacteria, Chlamydiae/Verrucomicrobia, and Fibrobacteres/Acidobacteria. Soon afterwards, Yin et al. [31] reported that butenolide could have a broad spectrum of anti-biofilm activity against both Gram-positive and Gram-negative pathogenic model species, including *Escherichia coli*, *Pseudomonas aeruginosa*, and methicillin-resistant *Staphylococcus aureus* (MRSA) with MBIC values ranging from 50 to 800 mg/L. This compound could effectively inhibit biofilm formation and eradicate mature biofilms of all the tested strains. The results of confocal laser scanning microscope (CLSM) and scanning electron microscope (SEM) showed that butenolide greatly removed biofilms by decreasing biofilm coverage and thickness and destroying biofilm matrix of microbial strains. Perhaps more significantly, it was demonstrated that butenolide had the potential to work with commonly used antibiotics for fighting biofilm infections due to its synergistic/enhancing anti-biofilm manner as a tetracycline enhancer. Based on these findings, butenolides should be considered as an attractive anti-biofilm candidate.

### 2.3. Terpenoids and Steroids

Some active anti-larval, anti-biofilm, and anti-bacterial terpenes (Figure 3) have been obtained from Streptomyces actinomycetes in recent years. The active AF diterpene compound **14**, namely lobocompactol, was obtained from a marine-derived actinobacterial strain *Streptomyces cinnabarinus* PK209 and found to have excellent anti-algal activity against the alga *U. pertusa* and the diatom *Navicula annexa* with EC_50_ values of 0.18 and 0.43 μg/mL and LC_50_/EC_50_ ratios of 46.2 and 71.6, respectively. Lobocompactol (**14**) also inhibited the growth of the fouling bacteria *Pseudomonas aeruginosa* KNP-5 and *Pseudomonas* sp. KNP-8 with MIC values of 66 μg/mL and 112 μg/mL, respectively [32]. Napyradiomycins belongs to a class of hybrid isoprenoids and/or meroterpenoids whose structures contain one or more terpene moieties bound to a non-terpenoid scaffold [33]. They are known for their antimicrobial and anticancer activities [34]. Until now, to our best knowledge, 61 napyradiomycin derivatives have been discovered and elucidated [33,34]. Recently, 12 napyradiomycins **15**–**26** isolated from the ethyl acetate extracts of *S. aculeolatus* strains PTM-029 and PTM-420 were reported as potential AF and anti-biofilm components by Pereira et al. [34]. Specifically, compounds **15**, **16**, **18,** and **24**, and mixtures of **17** and **21**, **19** and **20**, **22** and **25**, and **23** and **26** displayed AF activity against *Mytilus* *galloprovincialis plantigrade* larval settlements with EC_50_ values ranging from 0.10 to 6.34 µg/mL. Among these active napyradiomycins, the most effective ones were **15**, **24**, and **26**, showing EC_50_ value < 1 µg/mL (EC_50_ values of 0.66, 0.10, and 0.95 µg/mL, respectively), and the mixtures of compounds **22** and **25**, and compounds **23** and **26** with EC_50_ values of 0.73 µg/mL and 0.45 µg/mL, respectively. Importantly, all these compounds displayed low- or non-toxicity to the larvae of *M. galloprovincialis* with LC_50_/EC_50_ ratios >15 with the exception of compound **26**. Meanwhile, some of these AF napyradiomycins showed anti-biofilm activities against the five species of marine fouling bacteria, including *Marinobacter hydrocarbonoclasticus* DSM 8798, *Cobetia marina* DSM 4741, *Micrococcus luteus* DSM 20030 and ATCC 4698, *Pseudooceanicola batsensis* DSM 15984, and *Phaeobacter inhibens* DSM 17395. Among these compounds, napyradiomycin (**18**), the mixtures of compounds **19** and **20**, and compounds **22** and **25** were considered the most promising anti-biofilm components without inhibiting the growth of fouling bacteria assayed at the same concentration. Specifically, compound **18** showed significant biofilm inhibition (>90%) of *M. hydrocarbonoclasticus* at the lowest tested concentration of 0.98 µg/mL. The mixture of compounds **19** and **20** had no anti-bacterial activity at a concentration of 3.91 µg/mL, while it could prevent biofilm formation of *M. luteus* and *M. hydrocarbonoclasticus* DSM 8798 by 82.5 ± 5.1% and 43.8 ± 9.4%, respectively. The mixture of compounds **22** and **25** displayed anti-biofilm activity against *M. hydrocarbonoclasticus* was about 60% at all tested concentrations ranging from 0.98–15.60 µg/mL with no detected anti-bacterial activity, and completely abolished biofilm formation of *M. luteus* (100%) at the lowest concentration of 0.98 µg/mL but inhibited its growth by 70.5 ± 1.9%. Also, for the same mixture, significant inhibition of biofilm formation of *P. inhibens* (38.7 ± 0.7%) was observed at a concentration of 15.60 µg/mL, while not showing anti-bacterial effectiveness. The mixture of napyradiomycins **17** and **21** showed no anti-bacterial activity on these fouling microbes, but weak biofilm inhibition (>20%) for *M. luteus* and *C. marina*.

As shown in Figure 3, the chemical structure of napyradiomycins is characterized by the presence of a semi-naphthoquinone core, and herein all the reported napyradiomycins **15**–**26** consist of a prenyl unit attached at C-4a that is cyclized to form a tetrahydropyran ring and a monoterpenoid substituent at C-10a. The SAR analysis revealed that compounds **23**, **24,** and **26** containing the methyl group in the core structure at C-7 showed higher anti-larval activity. The 6-membered ring cyclized by the linear monoterpenoid subunit at C-10a in the most active compounds **22**–**26** may serve as an important functional group for enhancing the anti-larval activity of these napyradiomycin derivatives. Interestingly, three napyradiomycins **18**, **22,** and **25** with the highest anti-biofilm activity were obtained from the strain PTM-420, and contained a hydrogen atom at C-7. Conversely, napyradiomycins from PTM-029 generally had higher anti-biofilm activity with the methyl group at C-7. Additionally, napyradiomycins **22** and **25** stand out due to their broad-spectrum AF activity against macro- and micro-fouling organisms, which may be related with their chemical feature of a bromine substitute at C-16. Altogether, these above-mentioned natural anti-biofilm napyradiomycins should be further developed as promising anti-biofilm candidates that will not contribute to antibiotic/biocide resistance.

Only two steroids **27** and **28** (Figure 4) were isolated from a filamentous bacterium *Leucothrix mucor*, and found to prevent the spore settlement of the algae *U. pertusa* zoospores with EC_50_ values of 1.2 and 2.1 μg/mL as well as the growth of a biofouling diatom *Navicula annexa* with EC_50_ values of 5.2 and 7.5 μg/mL, respectively. The two compounds also exhibited anti-bacterial activity against the fouling bacterial strains *Pseudomonas aeruginosa* KNP-3 with MIC values of 32 and 56 μg/mL and *Alteromonas* sp. KNS-8 with MIC values of 66 and 90 μg/mL, respectively [35].

### 2.4. Anthraquinones

Two natural anthraquinones **29** and **30** (Figure 5) isolated from a rare actinobacterial strain *Kitasatospora albolonga* R62, together with their 10 commercial analogs were evaluated for antibiofilm activities against MRSA [36]. Among them, compounds **31**–**36** (Figure 5) could all effectively inhibit the formation of MRSA biofilms by >50%. It is noted that the inhibition rates of compounds **32** and **33** were up to 75.2% (±3.3%) and 96.4% (±0.3%). Also, both of these two compounds showed excellent eradication activity for preformed MRSA biofilms at concentrations of 200 and 50 mg/mL. The structure-activity relationship analysis of these anthraquinones suggested that the hydroxyl group attached at C-2 position of the anthraquinone skeleton should be considered as a key group for inhibiting biofilm formation at high concentrations, and the carboxyl group at the same C-2 position should play an important role in improving the eradication activity of these anthraquinones. The results of RNA sequencing indicated that the disruption of phosphate homeostasis of MRSA might be involved in the mode of action of compounds **32** and **33**.

### 2.5. Alkaloids

Eight bisindole alkaloids **37**–**44** (Figure 6), belonging to di (1*H*-indol-3-yl) methane (DIM) family, were isolated from a Red Sea ascidian-derived bacterium *Pseudovibrio denitrificans* UST4-50 [37]. All of them exhibited moderate to strong inhibitory activity against larval settlement of *B. amphitrite* with EC_50_ values ranging from 0.63 to 5.68 μg/mL, among which DIM (**37**) and 4-(di(1*H*-indol-3-yl) methyl)phenol (DIM-Ph-4-OH) (**40**) displayed excellent AF activity against larval attachment of *B. neritina* with EC_50_ values of 0.62 and 0.42 μg/mL, respectively, while their toxicity was very low with both LC_50_/EC_50_ ratios >60. Interestingly, the inhibitory ability of these two compounds for settlement of *B. amphitrite* cyprids was reversible, indicating special AF effects on macro-fouling invertebrates’ larvae. More encouragingly, the mixed paints containing 10–15% (*w*/*w*) DIM and basal ingredients showed comparable AF activity to the positive antifoulant Sea-Nine 211™ for 5 months or even longer in the field testing, which suggested that some potential AF DIMs might be considered as promising antifoulant candidates. The preliminary SAR of these DIMs was also discussed. It was found that the mother nucleus, di(1*H*-indol-3-yl) methylene, might be the functional group responsible for anti-larval activity, and the phenolic hydroxyl group linked to the Ph-C1′ would play a crucial role in increasing the anti-larval activity of these DIMs. Moreover, the supply issue of DIM has been easily solved by chemical synthesis or market purchases because of its simple structure. Further studies on AF characteristics of DIM and DIM-Ph-4-OH, such as the anti-algal activity against fouling algae, anti-biofilm activity against fouling bacteria, the evaluation of toxicity toward non-target organisms, AF mechanism using proteomic methods, and so on are in progress in our laboratory.

Two diketopiperazines (DKPs) (Figure 7), (6S,3S)-6-benzyl-3-methyl-2,5-diketopiperazine (bmDKP) (**45**) and (6S, 3S)-6-isobutyl-3-methyl-2,5-diketopiperazine (imDKP) (**46**), were obtained from a seaweed *Undaria pinnatifida*–derived *Streptomyces praecox* 291-11. Both bmDKP and imDKP could inhibit zoospore settlement of the seaweed *U. pertusa* with EC_50_ values of 2.2 and 3.1 μg/mL and the growth of the diatom *N. annexa* with EC_50_ values of 0.8 and 1.1 μg/mL, respectively. Compared to the low LC_50_/EC_50_ ratios of positive controls (being 2 and 6, respectively) toward the target algae, both compounds showed higher therapeutic ratios with LC_50_/EC_50_ ratios of 17.7 and 21 to *U. pertusa* and 263 and 120.2 to *N. annexa*, which indicated that these two compounds might be considered as non-toxic anti-algal compounds [38]. As a novel compound, Maipomycin A (MaiA) (**47**) (Figure 7), bearing a unique 2,20-bipyridine structure and an unusual oxime functionality, was isolated from a rare actinomycete strain *Kibdelosporangium phytohabitans* XY-R10. This compound showed strong anti-biofilm activities against Gram-negative bacteria including *Acinetobacter baumannii* ATCC 19606 and *Pseudomonas aeruginosa* ATCC 27853 with MBIC values of 8 and 32 mg/mL, respectively. More interestingly, it has demonstrated that this natural product might exert its antibiofilm activity through chelating irons [39].

### 2.6. Glycolipids, Benzenoids, Flavonoids, Polyketides, and Enzymes

Besides some bioactive compounds mentioned above, few of the glycolipids, benzenoids, flavonoids, and polyketides (Figure 8) derived from marine bacteria have also been evaluated for anti-biofilm and AF activities, and revealed a good level of effectiveness. A glycolipid surfactant **48** composed of glucose and palmitic acid was produced by a tropical marine strain of *Serratia marcescens* [40]. It showed anti-biofilm potential for preventing the adhesion of the marine biofouling bacterium *Bacillus pumilus* TiO1 with EC_50_ value of 6.25 μg/mL, and disrupting preformed biofilms of this culture in microtiter plates at concentrations ranging from 50 to 100 μg/mL. Interestingly, a marine-derived bacterial strain S6-15 that actually affiliates with the above-mentioned fouling species *B. pumilus*, could produce a novel 4-phenylbutanoic acid (**49**), which combated biofilm formation in all tested Gram-positive and Gram-negative species at concentrations ranging from 10–15 μg/mL [41]. Although dozens of flavonoids are widely distributed in the secondary metabolites of marine microorganisms and reported as bioactive compounds, herein only one flavonoid compound taxifolin (**50**) from a mangrove derived *Streptomyces sampsonii* PM33 was found to have AF activity [42]. In field experiment, marine surfaces of the test PVC panels coated with taxifolin (0.5 and 1.0 mg) did not have adherence of micro- or macro-fouling organisms even after 4 weeks. Further toxicity assays based on zebrafish models showed the lower toxicity of taxifolin at effective concentrations. These results indicate that taxifolin should be a promising candidate for the development of eco-friendly antifoulants. During the AF natural products project screening, the polyketide compound elasnin (**51**) highly produced by *Streptomyces mobaraensis* DSM 40847 exhibited strong biofilm inhibition activity against four marine bacterial Gram-positive strains *Staphylococcus aureus* B04, *S. hominis* N32, *S. arlettae* OM, and *Microbacterium esteraromaticum* N22 with MBIC_90_ and MBIC_50_ ranged from 2.5 to 5 µg/mL and 1.25 to 5 µg/mL, whereas three gram-positive strains *Idiomarina sediminum* N28, *Pseudoalteromonas* sp. L001, and *Escherichia coli* N57 with MBIC_90_ of 5 to 10 µg/mL and MBIC_50_ of 1.25 to 10 µg/mL, respectively [43]. The larval settlement of *Balanus amphitrite* was also effectively inhibited at concentrations above 12.5 µg/mL after 24 h exposure. Encouragingly, elasnin-based coatings showed significant performance in reducing the species richness and diversity of biofilms and the attachment of large biofouling organisms in the marine environment with a concentration of 2.5 wt% in the first 2 weeks. In addition, a low toxicity of elasnin towards *B. amphitrite* larvae was observed with a mortality rate of larvae around 10% under the concentration of 25 µg/mL compared with the control groups. These findings indicate that elasnin has a huge development potential for anti-biofilm and AF agents. So far, only a novel protease produced by a deep-sea bacterial strain *Pseudoalteromonas issachenkonii* UST041101-043, showed drastically strong AF effects on the settlement of *B. neritina* larvae with EC_50_ value of 0.5 ng/mL, and significant inhibition for larval settlement of the barnacle *B. amphitrite* and the bryozoan *Schizoporella* sp. at a very low concentration of 100 ng/mL [44].

## 3. Antifouling Natural Products from Marine Cyanobacteria

**Figure 9:**
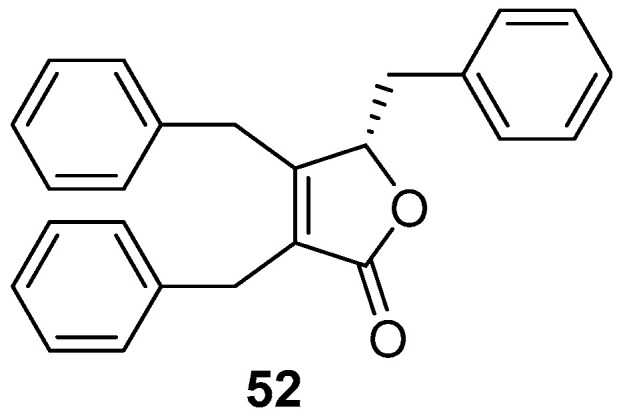
Chemical structures of the butenolide compound maculalactone A (**52**) from a marine cyanobacterium *Kyrtuthrix maculans*.

**Figure 10:**
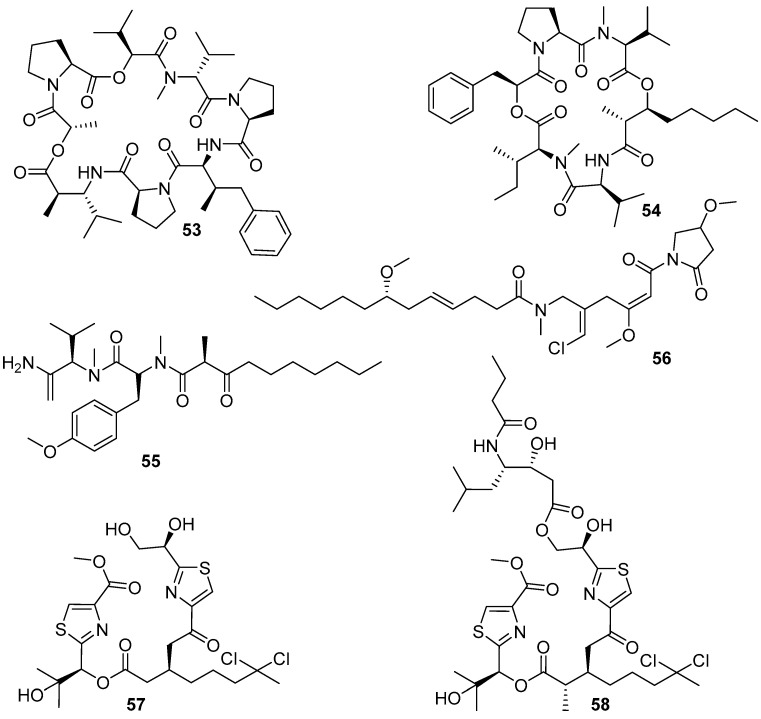
Chemical structures of **53**–**58** belonging to the polyketide–polypeptide structural family from marine cyanobacteria. Among them, compounds **53**–**56** were isolated from *Lyngbya majuscula*, and the dolastatin 16 (**53**) and lyngbyabellins **57** and **58** were isolated from *Okeania* sp.

Marine cyanobacteria are a bountiful source of novel bioactive metabolites, but the dearth of AF compounds isolated from cyanobacteria is still surprising nowadays. During the last two decades, only seven anti-larval secondary metabolites were obtained from marine-derived cyanobacteria [45,46,47,48,49], and there was no relevant report about anti-biofilm natural products. According to the chemical structures, these anti-larval are categorized into butenolides and polyketide–polypeptide structural families (Figure 9 and Figure 10).

### 3.1. Butenolides

A well-known butenolide compound maculalactone A (**52**) (Figure 9), as a “natural” marine AF agent from the marine cyanobacterium *Kyrtuthrix maculans*, exhibited excellent inhibitory activity against specific bivalve settlers at concentrations of 0.1% and 1% (*w*/*w*) in the preliminary field investigation, but was toxic to the naupliar larvae of the barnacles *B. amphitrite*, *Tetraclita japonica,* and *Ibla cumingii* with LC_50_ values ranging from 1.1–5.2 μg/mL [45]. And the stereochemistry of this natural product has been recently assigned by total synthesis [46].

### 3.2. Polyketide–Polypeptide Structural Compounds

The polyketide–polypeptide structural compound dolastatin 16 (**53**) (Figure 10), together with its three derivatives **54**–**56** (Figure 10), was isolated from the benthic filamentous marine cyanobacterium *Lyngbya majuscule*. All these four compounds showed anti-larval activity against the barnacle *B. amphitrite* larvae with EC_50_ values of 0.003, 10.6, 0.54, and 2.6 μg/mL, respectively. The most attractive dolastatin 16 has a good therapeutic ratio with LC_50_/EC_50_ ratio > 6000, and barnacle settlement on the dolastatin 16-treated plates was significantly reduced compared to negative controls at all the tested concentrations of 10.0, 1.0, 0.1, and 0.01 μg/mL, thus indicating its potential development prospects as a non-toxic antifoulant. Meanwhile, compounds **55** and **56** also displayed low toxicity with LC_50_/EC_50_ values of 167.7 and 77.6 except compound **54** with LC_50_/EC_50_ value of 6.7 [47]. Recently, natural dolastatin 16 was also found in the secondary metabolites of another marine cyanobacterium *Okeania* sp. [48], and the total synthesis of dolastatin 16 has been successfully achieved [50]. Both the synthetic and *Okeania* sp.-derived compounds showed similarly remarkable AF activity against *B. amphitrite* cyprid larvae to that of the previous *L. majuscula*-derived dolastatin 16, which further indicated that dolastatin 16 might serve as a potent AF compound for antifoulant candidates without concern about supply issues. Additionally, the strain of *Okeania* sp. could produce a series of AF lyngbyabellins, also belonging to the polyketide–polypeptide family, of which lyngbyabellins O (**57**) showed the most inhibitory effects on larval settlement of *B. amphitrite* with an EC_50_ value of 0.24 μg/mL, while its analogue lyngbyabellins P (**58**) was less active with an EC_50_ value of 0.62 μg/mL [48]. The investigation of SAR proposed that the acyclic form without a side-chain in compound **57** might be a functional structural framework for improving anti-larval activity of lyngbyabellins.

## 4. Anti-Biofilm and Antifouling Natural Products from Marine Fungi

**Figure 11:**
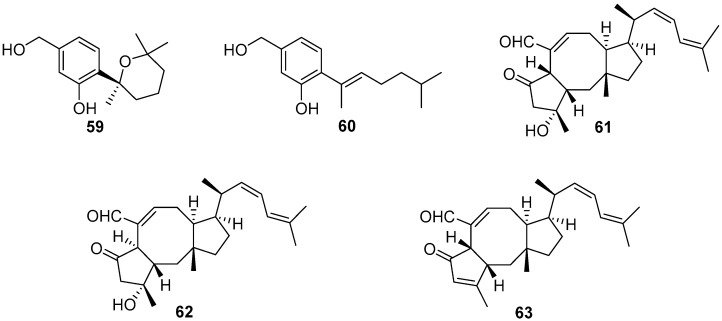
Chemical structures of bisabolane-type sesquiterpenoids **59** and **60** from *Aspergillus* sp., and sesterterpenes **61**–**63** belonging to the polyketide–polypeptide structural family from *Emericella variecolor*.

**Figure 12:**
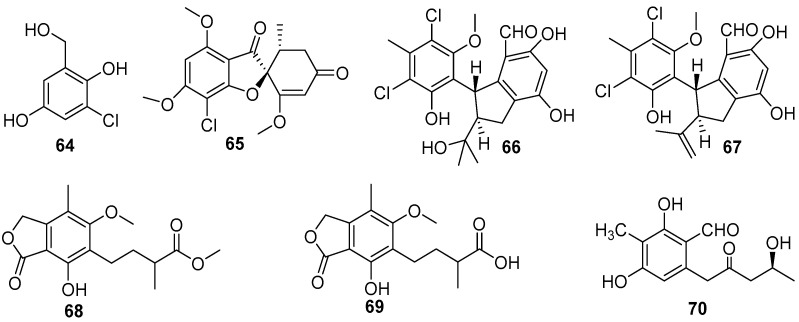
Chemical structures of chlorinated phenols **64**–**70** from marine fungi. Among them, compound **61** was isolated from *Ampelomyces* sp. UST040128, compounds **65** was isolated from *Sarcophyton* sp., and compounds **66** and **67** were isolated from *Pestalotiopsis* sp. ZJ-2009-7-6, and compounds **68**–**70** were isolated from strains *Penicillium brevicompactum* DFFSCS025 and *Aspergillus versicolor* SCSIO 41502.

**Figure 13:**
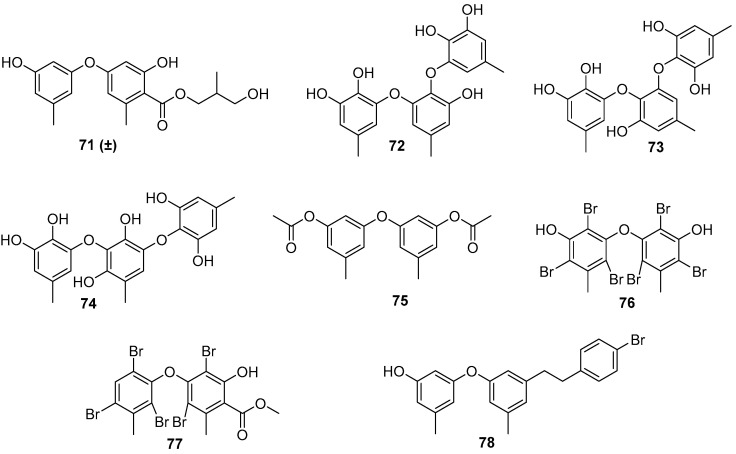
Chemical structures of phenyl ethers **71**–**78** from fungal strains of *Aspergillus*. Among them, compounds **71**–**74** were isolated from *A. versicolor* SCSIO 41502, and compounds **75**–**78** were synthetic derivatives of natural products from *Aspergillus* sp. XS-20090066.

**Figure 14:**
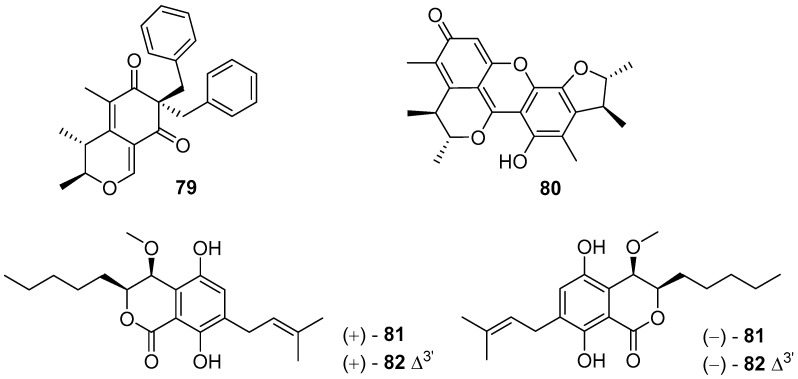
Chemical structures of benzylazaphilone analogue **79** from *Aspergillus* sp., compound **80** from *Xylariaceae* sp., and (±)-eurotiumides B (±**81**) and D (±**82**) from *Eurotium* sp. XS-200900E6.

**Figure 15:**
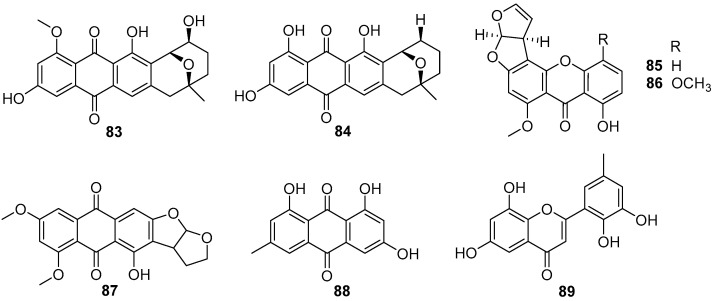
Chemical structures of anthraquinones and xanthones **83**–**86** from strains of *Aspergillus*, compound **87** from an unidentified fungal strain ZSUH-36, and anthraquinones **88** and **89** from *Penicillium* sp. SCSGAF 0023.

**Figure 16:**
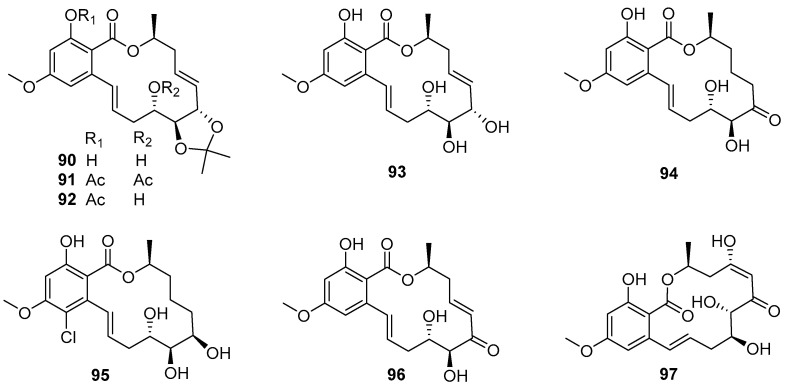
Chemical structures of 14-membered resorcylic acid lactones **90**–**97** from marine fungal strains of *Cochliobolus lunatus*.

**Figure 17:**
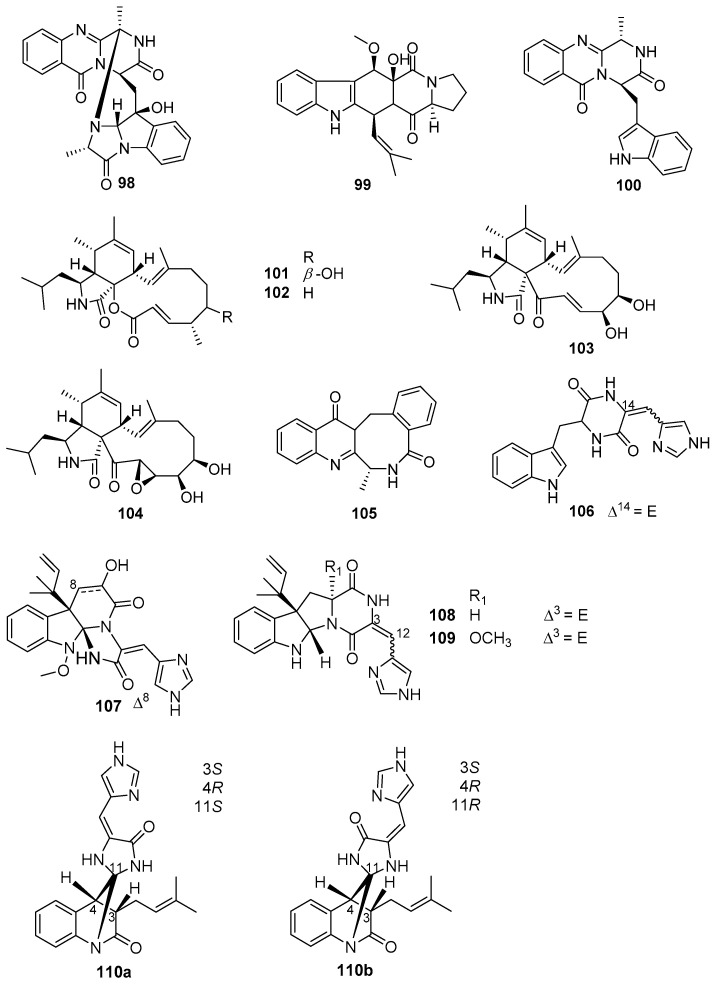
Chemical structures of alkaloids **98**–**110a**/**b** from marine fungal strains of *Aspergillus* and *Penicillium*. Among them, compounds **98**–**100** were isolated from *A. sydowii* SCSIO 00305, cytochalasins **101**–**104** were isolated from *A. elegans* ZJ-2008010, (+)-circumdatin F (**105**) was isolated from *A. alliaceus*, *A. westerdijkiae* DFFSCS013 and *A. westerdijkiae* SCSIO 05233, and indole alkaloids **106**–**109** and penispirolloid A (**110a**/**b**) were obtained from *Penicillium* sp. SCSIO 00258 and OUCMDZ-776, respectively.

**Figure 18:**
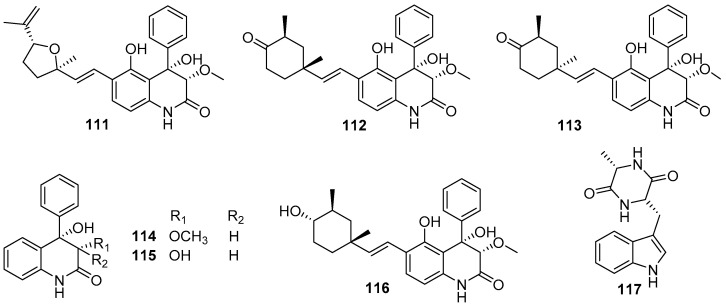
Chemical structures of alkaloids **111**–**116** from marine fungi *Scopulariopsis* sp., and the Cyclo-L-Trp-L-Ala (**117**) from *Eurotium chevalieri* MUT 2316.

**Figure 19:**
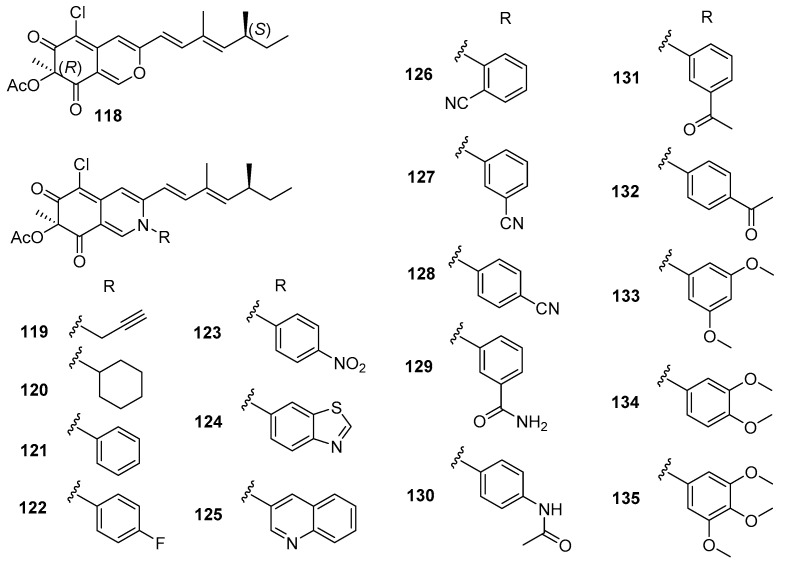
Chemical structures of the natural (+)-sclerotiorin (**118**) from a fungus *Penicillium sclerotiorum* CHNSCLM-0013 and its semisynthetic sclerotioramine derivatives **119**–**135**.

**Figure 20:**
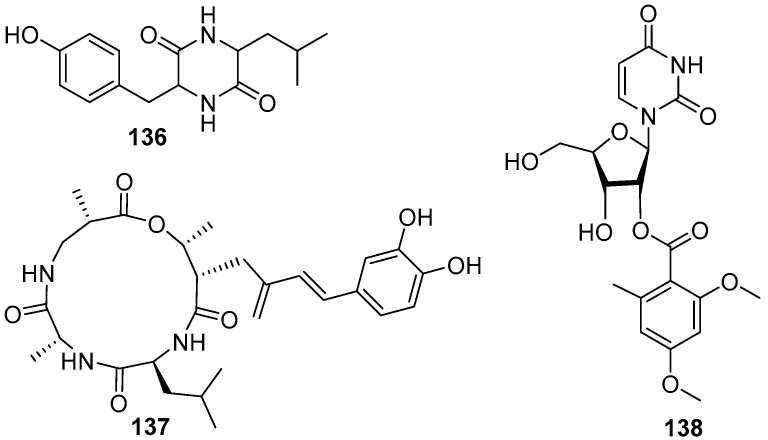
Chemical structures of the dipeptide *cis-cyclo* (Leucyl-Tyrosyl) (**136**) from *Penicillium* sp. F37, the cyclic tetrapeptide aspergillipeptide C (**137**) from *Aspergillus* sp. SCSGAF 0076, and the nucleoside diacetylkipukasin E (**138**) from *Aspergillus versicolor*.

Marine fungi are a main source of secondary metabolites with various biological activities and chemical structures, and offer more diverse anti-biofilm and AF natural products than marine bacteria and cyanobacteria. In this part, all natural products from marine fungi and their analogues are categorized into terpenoids, phenols, phenyl ethers, polyketides, alkaloids, amines, nucleosides, and peptides (Figure 11, Figure 12, Figure 13, Figure 14, Figure 15, Figure 16, Figure 17, Figure 18, Figure 19 and Figure 20).

### 4.1. Terpenoids

Four bisabolane-type sesquiterpenoids were isolated from a sponge-derived fungus *Aspergillus* sp., among which compounds **59** and **60** (Figure 11) could completely prevent larval settlement of *B. amphitrite* at a concentration of 25.0 μg/mL [51]. However, compound **59** was observed to have an obvious toxic effect on the larvae at its effective concentration. Three sesterterpenes named ophiobolin K (**61**), 6-epi-ophiobolin K (**62**), and 6-epi-ophiobolin G (**63**) (Figure 11) were obtained from the fermentation broth of a marine-derived fungus *Emericella variecolor*, and demonstrated to inhibit biofilm formation of *Mycobacterium* species to different degrees. Among them, ophiobolin K exhibited the best anti-biofilm activity against *M. smegmatis* and *M. bovis* BCG with MIC values of 1.58 and 3.15 μg/mL, respectively [52]. More importantly, ophiobolin K also has the ability to restore the antibacterial activity of isoniazid against *M. smegmatis* by preventing biofilm formation.

### 4.2. Phenols and Phenyl Ethers

Over the past two decades, dozens of benzenoids, especially phenols and phenyl ethers, have been isolated from marine fungi, and evaluated for AF activity, which led to the discovery of some effective anti-larval compounds. A series of phenol compounds were obtained from several species of four fungal genera, consisting of *Ampelomyces*, *Sarcophyton*, *Pestalotiopsis*, *Aspergillus,* and *Penicillium* [53,54,55,56]. Bioassay-guided isolation of crude extracts of *Ampelomyces* and *Pestalotiopsis* strains yielded four AF chlorinated phenols **64–67** (Figure 12) [53,54]. The compound 3-chloro-2, 5-dihydroxybenzyl alcohol (**64**) from *Ampelomyces* sp. UST040128 could inhibit larval attachment of *B. amphitrite* and *H. elegans* with EC_50_ values ranging from 3.192 to 3.812 μg/mL and from 0.672 to 0.78 μg/mL respectively. Meanwhile, its toxicity toward the *B. amphitrite* cyprids is very low with LC_50_ value of 266.682 μg/mL, yet it is highly toxic to the larvae of *H. elegans* with LC_50_ value of 2.642 μg/mL [51]. The amibromdole (**65**) was obtained from a soft coral-derived fungus *Sarcophyton* sp., and exhibited weak anti-larval activity against settlement of *B. amphitrite* larvae with EC_50_ value of 16.70 μg/mL [45]. Another two chlorinated phenols (±)-pestalachlorides E (**66**) and F (**67**) were isolated from *Pestalotiopsis* sp. ZJ-2009-7-6, showing excellent inhibition on settlement of *B. amphitrite* larvae with EC_50_ values of 1.65 and 0.55 μg/mL, and were not toxic to the cyprids with respective LC_50_/EC_50_ ratios >30.3 and >18.2 [47]. Three phenolic acids **68–70** (Figure 12), were obtained from two deep-sea-derived fungal strains *Penicillium brevicompactum* DFFSCS025 and *Aspergillus versicolor* SCSIO 41502, and these compounds showed anti-larval activity against *B. neritina* with respective EC_50_ values of 0.004, 0.006, and 3.40 μg/mL, but their toxicity was very low with all LC_50_/EC_50_ ratios >100 [55,56].

As for the marine fungi-sourced natural products, it should be noted that the fungal genus *Aspergillus* may be a potent source for the exploitation of AF phenyl ethers [56,57]. Five phenyl ether compounds **71**–**73** (Figure 13) from the deep-sea-derived fungus *A. versicolor* SCSIO 41502 were found to have anti-larval activity against settlement of *B. neritina* larvae with respective EC_50_ values of 1.28, 2.61, 5.48, 1.59, and 3.40 μg/mL, and LC_50_/EC_50_ ratios >100 [56]. Another six phenyl ethers, as characteristic secondary metabolites of *Aspergillus* sp. XS-20090066, together with their seven synthetic derivatives were demonstrated to have moderate to strong anti-larval activity against settlement of *B. amphitrite* larvae with EC_50_ values ≤ 14.11 μg/mL [57]. Among them, compounds **75**–**78** (Figure 13)were particularly promising, as they showed EC_50_ values ≤ 0.96 μg/mL and LC_50_/EC_50_ ratios > 20. Further studies of SAR indicated that the presence of the ester group and the introduction of bromine atoms at C-4 may be beneficial for enhancing anti-larval activity of these phenyl ethers.

### 4.3. Polyketides

In recent twenty years, polyketides, as one of the largest families of natural products from marine microorganisms, have provided many anti-biofilm and AF compounds with structural diversity, including benzylazaphilones, dihydroisocoumarins, anthraquinones, xanthones, and 14-membered resorcylic acid lactones (RALs) (Figure 14, Figure 15 and Figure 16). These compounds were mainly isolated from five fungal genera, including *Aspergillus*, *Xylariaceae*, *Penicillium*, *Cochliobolus*, and *Eurotium*.

#### 4.3.1. Benzylazaphilones and Dihydroisocoumarins

Aspergilone A (**79**) (Figure 14), a novel benzylazaphilone analogue from a gorgonian-associated fungus *Aspergillus* sp., could inhibit larval settlement of *B. amphitrite* with EC_50_ value of 7.68 μg/mL [58]. Dicitrinin A (**80**) (Figure 14), isolated from *Xylariaceae* sp., exhibited highly promising and non-toxic anti-larval activity against *B. neritina* larvae, with EC_50_ value of 1.76 μg/mL and a high LC_50_/EC_50_ ratio > 56 [59]. Four pairs of rare dihydroisocoumarin metabolites (±)-eurotiumides B (±**81**) and (±)-eurotiumides D (±**82**) (Figure 14) from a gorgonian-derived fungus *Eurotium* sp. XS-200900E6 were evaluated for AF activity against settlement of *B. amphitrite* larvae, and were all active in inhibiting larval settlement with EC_50_ values of 1.5, 0.7, 2.3, and 1.9 μg/mL, respectively. Importantly, their toxicities were low with LC_50_/EC_50_ ratios > 20 [60].

#### 4.3.2. Anthraquinones and Xanthones

Although anthraquinones and xanthonesthis as prominent secondary metabolites are isolated from cultural broth of some fungal strains, herein only eight compounds from two fungal genera of *Penicillium* and *Aspergillus* were investigated for their inhibitory effects on larval settlement of *B. amphitrite* [57,61,62,63]. Two *Aspergillus*-sourced anthraquinones averufin (**83**) and 8-*O*-methylnidurufin (**84**) (Figure 15) showed anti-larval activity against *B. amphitrite* with EC_50_ values of 2.03 and 3.39 μg/mL, respectively, but their toxicity was high with LC_50_/EC_50_ ratios >8.75 [57]. Other screenings of AF natural products from marine fungi yielded several anthraquinones and xanthones with strong to moderate inhibition abilities for settlement of *B. amphitrite* larvae, among which sterigmatocystin (**85**) and methoxysterigmatocystin (**86**) (Figure 15), isolated from *Aspergillus* strains, were the most highly active with EC_50_ values <0.125 μg/mL, but paralyzed the cyprid larvae at these effective concentrations [61]. Also, the xanthone 6,8-di-O-methyl versiconol (**87**) (Figure 15) from an unidentified mangrove endophytic fungus ZSUH-36, and two anthraquinones **88** and **89** (Figure 15) from a gorgonian-derived fungus *Penicillium* sp. SCSGAF 0023 showed anti-larval activity against *B. amphitrite* with respective EC_50_ values of 5.13, 6.10, and 6.70 μg/mL [62,63].

#### 4.3.3. 14-Membered Resorcylic Acid Lactones

Cochliomycins **90**–**95** (Figure 16), belonging to 14-membered resorcylic acid lactones (RALs) isolated from a gorgonian-derived fungus *Cochliobolus lunatus* HQ215514, were first demonstrated to have AF activity against larval settlement of *B. amphitrite* with EC_50_ values ranging from 1.2 to 17.9 μg/mL by Shao et al. [64]. Among these compounds, the most active one was cochliomycin A (**90**), with an EC_50_ value of 1.2 μg/mL, while its toxicity was low with LC_50_/EC_50_ ratio > 16.7. Proteomic analysis of *B. amphitrite* cyprids treated with cochliomycin A revealed the up- and down-regulation of proteins involved in the NO/cGMP pathway [65]. Later, some other similarly structural cochliomycins were obtained from a sea anemone-derived fungus *C. lunatus* TA26-46, and showed AF activity against *B. amphitrite* larvae with EC_50_ values ranging from 1.82 to 22.5 μg/mL, and the most active LL-Z1640-2 (**96**) (Figure 16) could inhibit larval settlement of *B. amphitrite* with EC_50_ value of 1.82 μg/mL and LC_50_/EC_50_ ratio > 50 [66]. As part of the ongoing research toward the AF cochliomycins from fungal strains of *C. lunatus*, another new compound cochliomycin G (**97**) (Figure 16) was found to have potent anti-algal activity against *Chlorella vulgaris*, *Chaetoceros socialis*, and *Navicula exigua* with EC_50_ values of 1.09, 0.92, and 0.61 μg/mL, respectively [67]. In addition, the results of SAR analysis suggested that the acetonide moiety, cis-enone moiety, and hydroxy groups should be essential for improving AF activity of these cochliomycins.

### 4.4. Alkaloids

Alkaloids, as a predominant member of marine microbes-derived AF natural products, were isolated from various species of marine fungi that are affiliated with the genera *Aspergillus*, *Penicillium*, *Scopulariopsis*, and *Eurotium*. Among these fungal strains, *Aspergillus* and *Penicillium* have been mostly well-studied [68,69,70,71]. Eight alkaloids **98**–**105** (Figure 17) produced by some strains belonging to the genus *Aspergillus* exhibited AF activity against larval attachment of *B. amphitrite* and *B. neritina* [68], of which compounds **98**–**100** from *A. sydowii* SCSIO 00305 displayed moderate anti-larval activity against settlement of *B. neritina* larvae with EC_50_ values ranging from 8.2 to 15.3 μg/mL. A series of cytochalasin alkaloids were obtained from a soft coral *Sarcophyton* sp.-derived fungus *A. elegans* ZJ-2008010, and evaluated for AF activity toward *B. amphitrite* larvae [69]. Among them, cytochalasins **101**–**104** showed anti-larval activity with EC_50_ values of 14.13, 5.59, 2.49, and 15.45 μg/mL, respectively. Analysis of SAR revealed that the electrophilic α,β-unsaturated ketone moiety and the double-bond at C-19 and C-20 in the most active aspochalasin D (**103**) might play an important role in increasing anti-larval activity of these cytochalasins. The benzodiazepine alkaloid (+)-circumdatin F (**105**) from *A. westerdijkiae* SCSIO 05233 was reported as an inhibitor for settlement of *B. amphitrite* larvae with EC_50_ value of 8.81 μg/mL [69]. Meanwhile, two strains SCSIO 00258 and OUCMDZ-776 of *Penicillium* were also found to produce some anti-larval secondary metabolites of alkaloids [68,71]. Among them, four indole alkaloids **106**–**109** (Figure 17) isolated from SCSIO 00258 showed anti-larval activity against *B. amphitrite* larvae with EC_50_ values ranging from 1.1 to 17.5 μg/mL, and the meleagrin (**107**) was considered as a potent AF candidate of ecofriendly antifoulants with a low EC_50_ value of 1.1 μg/mL and a high LC_50_/EC_50_ ratio > 22.6 [68]. In addition, a novel AF alkaloid penispirolloid A (**110a**/**b**) (Figure 17) possessing a unique spiro imidazolidinyl skeleton was isolated from a halotolerant fungus *Penicillium* sp. OUCMDZ-776, and also could prevent larval settlement of *B. neritina* with EC_50_ value of 2.40 μg/mL [71].

Besides those above-mentioned anti-larval alkaloids from *Aspergillus* and *Penicillium* strains, some other types of AF alkaloids were also obtained from various marine fungi. Several dihydroquinolin-2-one alkaloids were isolated from a gorgonian-derived fungus *Scopulariopsis* sp., all of which except aflaquinolone G displayed highly effective inhibition for larval settlement of *B. amphitrite*, with EC_50_ values of compounds **111**–**115** (Figure 18) being 0.007 pg/mL, 0.012 ng/mL, 0.001 ng/mL, 0.280 μg/mL, and 0.219 μg/mL, respectively [72]. Perhaps more notably, these active compounds were non-toxic with LC_50_/EC_50_ ratios ranging from 57 to 1200. Further chemical investigation of the fungus *Scopulariopsis* sp. led to the discovery of another new dihydroquinolin-2(1H)-one with a monoterpenoid side chain, scopuquinolone B (**116**) (Figure 18) [73]. This compound also showed outstanding anti-larval activity against the settlement of *B. amphitrite* larvae with EC_50_ value of 0.045 μg/mL and LC_50_/EC_50_ ratio of 222. Recently, the alkaloid cyclo-L-Trp-L-Ala (**117**) (Figure 18) from the sponge-associated fungus *Eurotium chevalieri* MUT 2316 was subjected to an enzymatic assay based on tyrosinase, which plays an important role in mussel byssus production. This compound displayed strong inhibition of tyrosinase with an LOEC value of 0.01 μg/mL, suggesting its potent anti-larval activity toward mussels [74].

### 4.5. Amines

The natural compound (+)-sclerotiorin (**118**) (Figure 19), isolated from a gorgonian coral-derived fungus *Penicillium sclerotiorum* CHNSCLM-0013, was reported as an AF compound for larval settlement of *B. amphitrite* with EC_50_ value of 5.6 μg/mL and LC_50_/EC_50_ ratio > 8.9 by Wei et al. [75]. In order to explore environmentally friendly AF amine derivatives, a series of 30 sclerotioramine derivatives were first synthesized, and evaluated for anti-larval activity against *B. amphitrite* larvae. A total of 25 semisynthetic derivatives showed anti-larval activity with EC_50_ values ranging from 0.47–18.2 μg/mL and LC_50_/EC_50_ ratios >2.7. Among these synthetic AF sclerotioramines, compounds **119–135** (Figure 19) showed potent anti-larval activities, which were stronger than that of the natural product (+)-sclerotiorin. The most active ones were the aliphatic amino-derivative **119** and the aromatic amino-derivative **134** with EC_50_ values of 0.94 and 0.47 μg/mL and LC_50_/EC_50_ ratios >53.2, indicating that both of them could be further developed as AF candidates as antifoulants. More interestingly, it should be mentioned that most of the potential anti-larval sclerotioramines belong to the aromatic amines except for compounds **119** and **120**, which suggest that the substitution of aromatic groups at N-2 should be crucial for increasing AF activities of these derivatives.

### 4.6. Peptides and Nucleosides

A dipeptide compound *cis-cyclo* (Leucyl-Tyrosyl) (**136**) (Figure 20) was isolated from a marine fungus *Penicillium* sp. F37, and found to be capable of inhibiting ~80% biofilm formation of a pathogenic bacterium *Staphylococcus epidermidis* at the concentration of 1.0 mg/mL, while the growth of the target strain was not interfered [76]. At the same time, a cyclic tetrapeptide aspergillipeptide C (**137**) (Figure 20), isolated from a fungus *Aspergillus* sp. SCSGAF 0076 associated with a gorgonian *Melitodes squamata*, displayed anti-larval activity against *B. neritina* with EC_50_ value of 11 μg/mL and LC_50_/EC_50_ ratio > 25 [77]. Only one AF nucleoside compound diacetylkipukasin E (**138**) (Figure 20) was obtained from marine microorganisms. This compound was isolated from a gorgonian *Dichotella gemmacea*-derived fungus *Aspergillus versicolor*, and displayed weak inhibitory effect on settlement of *B. amphitrite* larvae with EC_50_ value of 22.5 μg/mL [78].

## 5. Conclusions and Looking Ahead

In the last two decades, few studies on anti-biofilm natural products from marine microorganisms have been reported, except for the twelve compounds belonging to the five chemical types described in this review. Among these anti-biofilm natural products, the most effective ones are butenolide (**7**) and terpenoid compounds napyradiomycins **18–20** and 2**5,** with the exception of ophiobolin K (**61**). They are all considered highly promising AF compounds for further development of antifoulant candidates. As mentioned at the beginning, biofilms play essential roles in biofouling of macro- and micro-foulers because of inducing the settlement of invertebrates’ larvae and algae spores, which indicates that some effective anti-larval/anti-algal compounds may possess a promising potential for anti-biofilm agents. Herein, a total of 112 important anti-larval and anti-algal natural products, together with their 26 synthetic analogues, are shown from Figure 1, Figure 2, Figure 3, Figure 4, Figure 5, Figure 6, Figure 7, Figure 8, Figure 9, Figure 10, Figure 11, Figure 12, Figure 13, Figure 14, Figure 15, Figure 16, Figure 17, Figure 18, Figure 19 and Figure 20. According to Qian et al. [17] and Fusetani et al. [18], a compound with an EC_50_ value of <5 μg/mL and a LC_50_/EC_50_ ratio of >50 can be considered a highly promising candidate for environmentally benign AF agents, but those with lower AF concentrations and higher therapeutic ratios are more desirable. As such, some significantly efficient and non-toxic anti-larval/anti-algal compounds from marine microbes, including the following: *Streptomyces*-derived butenolides and terpenoids, *Pseudovibrio*-derived bisindole alkaloids, *Pseudoalteromonas*-derived protease, cyanobacteria-derived polyketide–polypeptide structural compounds, fungi-derived phenols and phenyl ethers, *Xylariaceae*-derived benzylazaphilones and *Cochliobolus lunatus*-derived 14-Membered RALs belonging to the polyketides, *Scopulariopsis*-derived alkaloids, and semisynthetic sclerotioramines based on *Eurotium chevalieri*-derived amine, are worthy of exploitation for their potential as anti-biofilm and AF candidates. Undoubtedly, more and more AF natural products from marine microorganisms will be discovered in the future due to their great AF applications in the medical and marine industries. It is a feasible and time-saving way to get a certain amount of anti-biofilm agents by screening for anti-biofilm activity of marine microbes-derived AF natural products.

The main challenges for the development of anti-biofilm/AF compounds have been highlighted in previous reviews [2,7,8,12,13,14,15,16,17,18,19,20]. As we have been advancing our knowledge of how to bioprospect for potent anti-biofilm/AF from marine microorganisms, a few of the issues remaining in current research or application studies should be addressed in future research. First, although the so-called “continuous supply” is one of the major factors that warrants microbe-derived anti-biofilm/AF agents for biological assays and preclinical/clinical/field trials, a few promising and effective natural products have complex structures and low fermentation yields, which hinders their further development. To solve the bottleneck problem of supply, genetic engineering technology can be employed for the culture and fermentation of microbes to increase the yield of target substances, and synthetic/semi-synthetic strategies could also be developed to satisfy the demand for large quantities of compounds for application in commercial or medicinal areas. Second, for those potent anti-biofilm/AF natural products based on the evaluation through biofilm assays/antifouling activity evaluation platforms in laboratories, further in vivo outcomes/field experiments should be performed since the results could be very different. Third, the basic mechanistic aspects of how anti-biofilm/AF compounds exert their activity should be well understood. Over the past twenty years, a number of genes and proteins that may be involved in biofilm formation or fouling of invertebrate larvae and algae have been identified through genomic and proteomic approaches [22,27,29,65,79,80,81,82]. In the near future, more efforts need to be invested to decipher the specific signaling pathways that are either conserved, or unique to the biofilm formation/eradication, and settlement/anti-settlement processes, which will facilitate the introduction of anti-biofilm/AF to the market. Fourth, comprehensive toxicity tests need to be established. For anti-biofilm agents, before advancing to costly animal models, simple nematode models can be used for the preliminary toxicological analysis [82,83,84,85,86,87,88,89]. *Caenorhabditis elegans*, as a tiny model organism, is often used in toxicity studies of small molecules owing to its complete genome, short lifespan, and simple physiology [84,90,91]. For antifouling compounds, their acute toxicity to larvae of model macro-foulers, chronic toxicity to some model organisms (such as zebrafish), as well as their degradation kinetics of in the marine ecosystem need to be evaluated before commercialization. Finally, it has been demonstrated that bacterial quorum sensing (QS) plays an important role in biofilm formation and infectious processes [2,14,15]. Hence, development of efficient and safe anti-biofilm agents via interfering with QS to inhibit biofilm formation and reduce virulence without inhibiting basic growth of bacteria may be a promising solution to treat bacterial infections.

Last but not least, in addition to purified compounds, dozens of crude extracts from marine microbes were also found to have good anti-biofilm/AF activity in the past decades, which provided potential sources for further exploitation as novel anti-biofilm/AF compounds. For example, the crude extract of an actinobacterial strain *Glycomyces sediminimaris* UTMC 2460 caused 93.2% and 71.4% reduction in biofilm biomass of two fouling bacterial strains *Kocuria* sp. UTMC 2449 and *Mesorhizobium* sp. UTMC 2518 with MIC value of 100 µg/mL, respectively, and it was not toxic toward *Artemia salina* larvae [92]. According to Viju et al. [93], the coating developed with 15.8 and 27.5% (*w*/*w*) crude extract of the bacterium *Pseudomonas putida* showed a significant reduction in the recruitment of fouling organisms over a period of 50 days under natural marine conditions. For these promising and effective crude extracts of marine bacteria mentioned above, it is necessary to carry out further investigation of separation, purification, and chemical identification of the active antibiofilm/AF compounds present in these extracts.

Overall, it is well recognized that AF natural products from marine microorganisms could provide a promising source for anti-biofilm compounds. Nowadays, there are still many obstacles restricting the development of anti-biofilm/AF agents in medical and industrial applications. To meet these major issues as mentioned in this review, collaborative endeavors involving natural products chemistry with organic chemistry, microbiology, pharmacology, and biology will help to build up a fairly systematic research system, and facilitate an increase in marine microorganisms-derived natural products reaching the clinical trials/market as anti-biofilm therapeutics/antifoulants.
